# Cancer as a Disease of Development Gone Awry

**DOI:** 10.1146/annurev-pathmechdis-031621-025610

**Published:** 2023-10-13

**Authors:** Ben Z. Stanger, Geoffrey M. Wahl

**Affiliations:** 1 Division of Gastroenterology, Department of Medicine, Abramson Family Cancer Research Institute, and Department of Cell and Developmental Biology, Perelman School of Medicine at the University of Pennsylvania, Philadelphia, Pennsylvania, USA;; 2 Gene Expression Laboratory, The Salk Institute for Biological Studies, La Jolla, California, USA;

**Keywords:** cancer, embryonic development, stem cells, plasticity, reprogramming, cell state

## Abstract

In the 160 years since Rudolf Virchow first postulated that neoplasia arises by the same law that regulates embryonic development, scientists have come to recognize the striking overlap between the molecular and cellular programs used by cancers and embryos. Advances in cancer biology and molecular techniques have further highlighted the similarities between carcinogenesis and embryogenesis, where cellular growth, differentiation, motility, and intercellular cross talk are mediated by common drivers and regulatory networks. This review highlights the many connections linking cancer biology and developmental biology to provide a deeper understanding of how a tissue’s developmental history may both enable and constrain cancer cell evolution.

## INTRODUCTION AND HISTORICAL PERSPECTIVE

Early in the nineteenth century, with the microscope as the only instrument to analyze tumor composition, embryologists, developmental biologists, surgeons, and pathologists hypothesized that malignant cancers arose from embryonic cells, or cells resembling those found in the embryo. As there are detailed historical reviews concerning observations made by these early researchers investigating cancer from diverse disciplines with rudimentary tools (1–3), we only briefly refer to these early studies to set the stage for more contemporary perspectives. Remarkably, elements of the early researchers’ hypotheses still hold true for some neoplasms; consequently, reprogramming of cells to a developmentally plastic state like that in the embryo is emerging as a common observation in many malignant cancers.

Around the same time that the links between development and cancer were first proposed, Matthias Schleiden and Theodor Schwann proposed the cell doctrine, which was the initial description that animals and plants are composed of smaller units called cells. This led to the hypothesis by Johannes Müller suggesting that malignancies could be categorized by the types and proportion of cell types they contained (4). The father of pathology, Rudolf Virchow, is credited with the important concept of omnis cellula e cellula (all cells come from cells), but it took the work of developmental biologists such as Müller, Robert Remak, and others to demonstrate that organs derive from cells of the same type, leading to the conclusion that cancers originate from cells of the organ in which they are found (4).

Even before the cellular theory of cancer was proposed or accepted, the gynecologic surgeon Joseph Récamier observed in 1829 that small cells that appeared to be of an early developmental state were present in some tumors (4). This led him to suggest that primitive germ cells might be the source of some tumors. The potential connection between proliferative, undifferentiated embryonic cells and cancer evolved into an embryonic rest theory for the origin of cancer, which advanced over the mid-nineteenth century and was summarized in 1874 by the surgical pathologist Francesco Durante (5, p. 95):

Elements which have retained their…embryonal characteristics in the adult organism, or that have regained them through some chemico-physiologic deviation, represent…the generative elements of every tumor variety and specifically those of a malignant nature. Such elements may remain enclosed within matured tissues for many years, giving no indication of their presence, until an irritation—a simple stimulus suffices—rekindles their vital cellular activities.

Here, Durante envisions two possible routes to cancer through embryonic intermediates: (*a*) an embryonic cell arrested in its differentiation that persists in the adult, or (*b*) an adult cell that could reacquire embryonic characteristics. These ideas still resonate today as biologists consider whether cancers arise from cells that retain embryonic features, such as stem cells, or whether they emerge by dedifferentiating to an embryonic state resulting from Durante’s hypothesized persistent irritation.

Other parallels between early embryonic development and malignancy were coming to light around the same time. The British developmental biologist John Beard noted similarities between the trophoblast and invasive tumors, since it is the trophoblast that invades the uterus, grows extensively, recruits a blood supply, and suppresses the maternal immune system to enable fetal growth (6). This led Beard to suggest that aggressive tumors could derive from displaced trophoblastic cells. While the details of Beard’s model have not been sustained, the principle that cancer cells develop the invasive properties needed to relocate to distant tissue sites, establish a circulation, and suppress immune recognition is reflected in our current understanding of the metastatic cascade (7).

**Stem cells:**

individual cells with the properties of multipotency (the ability to give rise to multiple cell types) and self-renewal (the ability to give rise to more stem cells)

**Trophoblast:**

the outer cells of an early mammalian embryo from which the placenta eventually forms

**Metastatic cascade:**

the series of events (invasion, vascular intravasation, survival in the bloodstream, vascular extravasation, and colonization) that enable tumor cells to spread from a primary site and grow in a distant organ

Taken together, work from the early nineteenth century to the beginning of the twenty-first century provided compelling evidence for cancers as diseases of development gone awry. Until recently, Durante’s proposition that cell state changes (i.e., dedifferentiation and/or reprogramming) constitute a critical step in cancer initiation remained outside of the mainstream, as oncogenic drivers were felt to be sufficient for oncogenic transformation (8). In this review, however, we discuss how new, real-time tumor tracking approaches are revealing that an interplay between adult cell states and those resurrected from embryonic life underlies most cellular and molecular hallmarks of cancer, supporting the inclusion of cell state plasticity as a newly introduced cancer hallmark (9).

## GROWTH CONTROL IN CANCER AND DEVELOPMENT

Rapid cell proliferation may be a defining feature of most cancers, but the growth rate of a human embryo during the second trimester equals or exceeds that of even the most aggressive malignancies (10). The cell division machinery is the same for both processes, and yet the embryo ultimately slows its growth whereas this rarely occurs in tumors. The mechanisms by which normal tissues suspend growth at the end of development serve as potent barriers to malignant transformation, and therefore understanding this process is likely to inform new anticancer strategies.

It is important, at the outset, to distinguish cellular growth and proliferation programs from size-control programs. The former processes encompass well-studied molecular pathways by which cells accumulate biomass (via growth programs), proliferate (via cell cycle and mitotic programs), and evade death (via survival programs), facilitating the growth of both normal organs and tumors derived from them. We now have a deep understanding of the transcriptional control mechanisms, signaling pathways, enzymes, and motor proteins that facilitate tissue expansion, all of which actively participate in cancer. By contrast, the mechanisms regulating tissue size have puzzled biologists for more than a century, for they concern the organismal-level blueprints that provide oversight of the more mechanical growth and proliferation programs. Size control ensures that an elephant is “elephant-sized,” that the limbs on both sides of the body are roughly the same length, and that growth slows at the appropriate point in development. Yet despite years of study, and the obvious relevance of size control to cancer, we still have a poor understanding of the evolutionarily programmed guidelines governing size at the tissue level.

One hint regarding size-control mechanisms comes from a classical dichotomy in embryology: the extent to which a developmental process is controlled autonomously (nature) or regulated (nurture). Cell fate decisions in the embryo are controlled by a balance between regulated responses to external forces and autonomous, intrinsic cellular proclivities. This balance also plays out in the control of mammalian tissue size, where some organs achieve their final size through regulatory feedback loops, while other organs employ autonomous growth programs that are minimally dependent on external signals (11). Similarly, cancer reflects an interplay between dysregulated environmental signals (e.g., growth factors) and dysregulated cell intrinsic pathways arising during cancer progression. Alterations in oncogenes and tumor suppressor genes endow cancer cells with a degree of independence from external growth signals. But a cell’s epigenetic fingerprint, the landscape resulting from its developmental history, also guides and constrains cellular growth properties, a topic discussed below in our consideration of the cancer cell of origin.

**Cell state plasticity:**

the ability of a cell to change its phenotype, identity, or differentiation trajectory

**Cell fate:**

the phenotypic outcome of cellular differentiation; typically connotes what will happen to a cell if left unperturbed, as a default (evaluated most accurately by lineage tracing, not by transplantation)

**Oncogenes:**

a class of genes that normally function to regulate cell growth, proliferation, metabolism, or other processes that, when mutated, promote tumorigenesis

**Tumor suppressor genes:**

a class of genes that prevent tumorigenesis by inhibiting cell growth and proliferation, inducing cell death, or preventing acquisition of cell state plasticity and that may be activated in response to genome damaging or stressful conditions

### Cell Competition

Cell competition, an evolutionarily conserved mechanism governing tissue size in embryos, has emerged as an important feature of many cancers. Competition is conventionally viewed as a passive process, a Darwinian type of natural selection wherein differences in the fitness of two populations lead to selective expansion, over time, of the more fit population at the expense of the less fit population (12). But in the context of embryonic development, cell competition has a more specific meaning: it describes an active process whereby more fit cells do not merely outgrow their less fit neighbors; they kill them.

Originally identified in *Drosophila*, cell competition involves intercellular comparisons of cellular vigor or fitness (13). While molecular mechanisms are still being resolved, competitive interactions involve comparisons of growth or metabolic properties between cells, resulting in two outcomes: (*a*) a determination of which cells have greater fitness (called winners) and which have lesser fitness (called losers), and (*b*) the elimination, via apoptosis or other means, of the loser cell by the winner cell. This induced cell death thus makes room for the cells with more advantaged growth properties without causing an overgrowth of the tissue compartment. The phenomenon has been most clearly demonstrated through the study of mutant cells in the fly wing imaginal disc, where overexpression of cell growth regulators such as *Drosophila* Myc (dMyc) leads to the formation of giant mutant clones but a preservation of the overall wing size (14). Cell competition also operates in mammalian embryogenesis, where it is involved in the selection of fit cells during the development of the blastocyst and the epiblast (15).

Importantly, this embryonic growth control mechanism also participates in malignant progression (16), where it can have either tumor-promoting or tumor-suppressing activities (17). Competitive behavior promoting tumor development has been well documented in the intestine, where oncogenic mutations in *APC*, *KRAS*, or *PIK3CA* facilitate tumorigenesis through both tumor cell-intrinsic effects on cell growth and non-cell-autonomous effects on neighboring cells. Competitive cell interactions have their most important effects during the earliest stages of (pre)malignant progression, a period that is particularly difficult to study. In that setting, the molecular paths taken by cells as they transform are especially important, as the presence or absence of competition is likely to determine how readily an early lesion can be detected. For example, it is possible that mutations that engender cell competition will allow a cell’s clonal offspring to spread laterally within an epithelial layer, replacing the normal tissue in what is commonly known as a field effect. Mutations that fail to result in a competitive interaction, by contrast, may be unable to replace the normal epithelium through lateral growth and hence be forced to grow in a different plane (i.e., perpendicular to an epithelial sheet). This may explain the difference between intestinal adenomas that grow into the luminal space as visible polyps as opposed to the flat adenomas that expand laterally within the epithelial layer, making them much harder to detect.

**Adenomas:**

premalignant lesions arising in an epithelial tissue in which one or more mutations lead to abnormal growth properties

### Oncogenes and Tumor Suppressor Genes

Cancer results from genetic and epigenetic alterations acquired during tumor progression that dysregulate cell growth, survival, and proliferation pathways. Some cancer-associated genes, such as *TP53* (the so-called guardian of the genome), are broadly mutated across cancer regardless of tissue type. But most genes with a role in cancer have a narrower spectrum of activity. One striking observation from The Cancer Genome Atlas, the comprehensive molecular catalog of thousands of human tumors, is that some mutations occur in patterns that closely track with a tumor’s developmental history (i.e., tissue of origin) (18). Mutations in the *KRAS* oncogene, one of the most highly mutated oncogenes in human cancer, are found almost exclusively in carcinomas arising from endoderm derivatives (pancreas, lung, liver, and intestines) (19). In other cases, the mutational spectrum does not track with a particular embryonic lineage but still exhibits striking tissue specificity; mutations in isocitrate dehydrogenase 1 (*IDH1*) and *IDH2*, for example, are found in brain tumors, cholangiocarcinomas, and leukemias but are rare in other tumor types (20). Indeed, mutations in the same gene may be either tumor promoting or tumor suppressing depending on tissue type, as exemplified by the finding that leukemias are frequently associated with gain-of-function mutations in *NOTCH1* or *NOTCH2*, whereas squamous cell carcinomas are frequently associated with loss-of-function mutations in the same gene(s) (21). These contrasting activities may be related to the fact that NOTCH signals promote proliferation in lymphocytes and differentiation in the skin (22, 23), explaining how activation of the pathway might be oncogenic in the former and tumor suppressing in the latter.

The molecular basis underlying this relationship between gene and tissue—molecular tropism—persists as one of the most important unsolved questions in cancer biology. The problem is made even more puzzling by the fact that mutational patterns bear no obvious connection to developmental need. For example, null mutations in *KRAS* are associated with defective hematopoiesis, heart defects, and motoneuron death but no overt developmental abnormalities in organs where it plays a major oncogenic role (24, 25). The strong association between tumor type and genotype indicates that a cell’s developmental history dictates the spectrum of oncogenic activity to which it is susceptible, a concept supported by large multiomic studies (26). Stated otherwise, cancer can be viewed as a manifestation of the right genes being altered in the right cells at the right time. The molecular logic guiding this selectivity remains unknown but may be related to the different metabolic requirements that cells from different tissues must meet during malignant progression (27).

**Molecular tropism:**

the observation that certain mutant oncogenes and tumor suppressor genes are found in only certain tumor types

**Hybrid cell:**

a cell exhibiting characteristics of more than one cell type and increasingly found as an intermediate in metaplasia and during cancer progression

## STEM CELL BIOLOGY

Stem cells, specialized subpopulations that self-renew and differentiate according to their microenvironment(s), constitute important intermediates in embryonic development. Links between stem cell biology and cancer biology emerged from the observation that malignant tumors arose after transplanting individual (normal) germ cells from the genital ridge into the testes of normal 129/SIJ/129 mice. Further investigation of these tumors (teratomas) revealed cells of multiple tissue lineages at different stages of differentiation. By contrast, no teratomas arose following the transplantation of genital ridge cells from genetically sterile S1/S1 fetuses, which lack germ cells. Furthermore, teratogenesis is suppressed by transplanting cells from a teratoma into a normal blastocyst (28). Together, these studies powerfully demonstrated that a normal germ (stem) cell in a compatible microenvironment can induce tumorigenesis and that tumorigenesis is suppressed by a normal microenvironment (29, 30).

These landmark studies provided clear evidence that certain kinds of tumors could arise from stem cells. Furthermore, they demonstrated the importance of both the seed and the soil for tumor initiation and suppression, supporting Durante’s prediction that cancer could arise by alterations in the environment in which an initiated cell is situated. As discussed below, mutations in cancer genes, lineage specifying transcription factors, and epigenetic regulators can increase the probability of converting differentiated adult cells into plastic, hybrid cell states that, in the setting of a permissive (e.g., inflammatory) microenvironment, result in cancer.

### The Hematopoiesis Paradigm

The principles of stem cell biology were initially developed through bone marrow transplantation experiments performed in the aftermath of the Second World War. Rodent studies revealed the existence of single bone marrow cells that could rescue lethally irradiated animals by differentiating into both lymphoid and myeloid lineages; moreover, the cells retained this ability over multiple transplant generations (31, 32). These observations established the existence of hematopoietic stem cells (HSCs) as cells able to (*a*) generate the multiple cell types present in a corresponding adult organ (multipotency) and (*b*) make more of themselves (self-renewal). This created a paradigm for the complementary approaches adopted by cancer biologists studying diverse solid tumors.

**Progenitor cells:**

cells possessing the properties of multipotency and self-renewal but whose potential and ability to generate more progenitor cells is less than that of a stem cell

**Lineage tracing:**

a cell-marking method allowing the determination of cellular ancestries, starting from a group of cells or individual cells

**Barcoding:**

a lineage tracing method in which individual cells are marked with exogenous pieces of DNA bearing unique sequences (barcodes) allowing for high-resolution tracking of cell fate(s)

**Cell potential:**

the range of fates available to a cell following a perturbation (e.g., stimulation, relocation, damage, or transplantation)

Early analyses suggested that HSCs are rare and divide infrequently, a finding that was interpreted as an evolutionary design to limit opportunities for mutation accumulation. As the fully differentiated cell types of the hematopoietic system have limited life spans and divide infrequently, homeostatic control was inferred to derive from proliferative progenitors resulting from the asymmetric division of an HSC; such progenitors, in turn, were presumed to produce increasingly lineage restricted progeny. This led to what has become the standard hierarchical model for hematopoiesis in which an HSC resides at the apex of a tree-like lineage, with bipotential progenitors designed to make abrupt, alternative fate choices at each branch point, ultimately yielding the fully differentiated cells that execute the essential functions of the lymphoid, myeloid, and erythroid systems (31).

This hierarchical model made strong predictions that have now been tested with more precise methods. Consistent with the model, HSCs are rare, largely dormant (dividing, on average, every two months), and capable of self-renewal (33). (As we will see later, not all stem cells are dormant.) HSCs may comprise multiple cellular phenotypes, likely influenced by local microenvironments, as some cells with HSC properties divide only five times during a mouse’s life span (34, 35); hence, there even may be a subhierarchy within the HSC population. Importantly, quiescence may not prevent against mutation accumulation since whole genome sequencing analyses of dividing and nondividing cells indicate similar rates of mutation accumulation (36).

Recent studies of hematopoiesis suggest that differentiation involves gradual transitions at the transcriptome level, mediated by steady rather than abrupt changes in the ratios of lineage-specifying transcription factors. In line with this view, it has been proposed that intermediates in differentiation should not be referred to as bipotential but rather as fate limited (37). Even the HSCs at the apex of the hierarchy are not restricted to one path of differentiation. Rather, their fate can be profoundly impacted by their microenvironment, presumably because they coexpress transcription factors associated with different lineages (38). This property is likely to be a general characteristic of stem and progenitor cells. For example, the embryonic progenitors of the basal and luminal lineages of the adult mammary gland also differentiate by gradual transcriptomic changes after birth, and embryonic mammary stem cells coexpress markers and transcription factors found in both basal and luminal cells (39, 40). This coexpression presumably enables cells to rapidly differentiate when exposed to the appropriate microenvironment or changes in cell–cell contacts. This might provide one explanation for why it has been so difficult to define a unifying stem cell transcriptome or proteome.

The transplantation assays required to reveal stem cell properties of HSCs use high-dose radiation to eliminate resident stem cells prior to introducing fresh cells. This perturbs the microenvironment in which HSCs normally reside (the niche) as a part of the method designed to assess function. This is an important consideration, as studies in many organs and organisms have shown the importance of the niche for stem cell function (41, 42). Transplantation into an empty bone marrow therefore measures cellular function under extreme conditions, reflecting the state engendered by chemotherapy or the transplantation assay itself rather than normal homeostasis.

**Figure 1:**
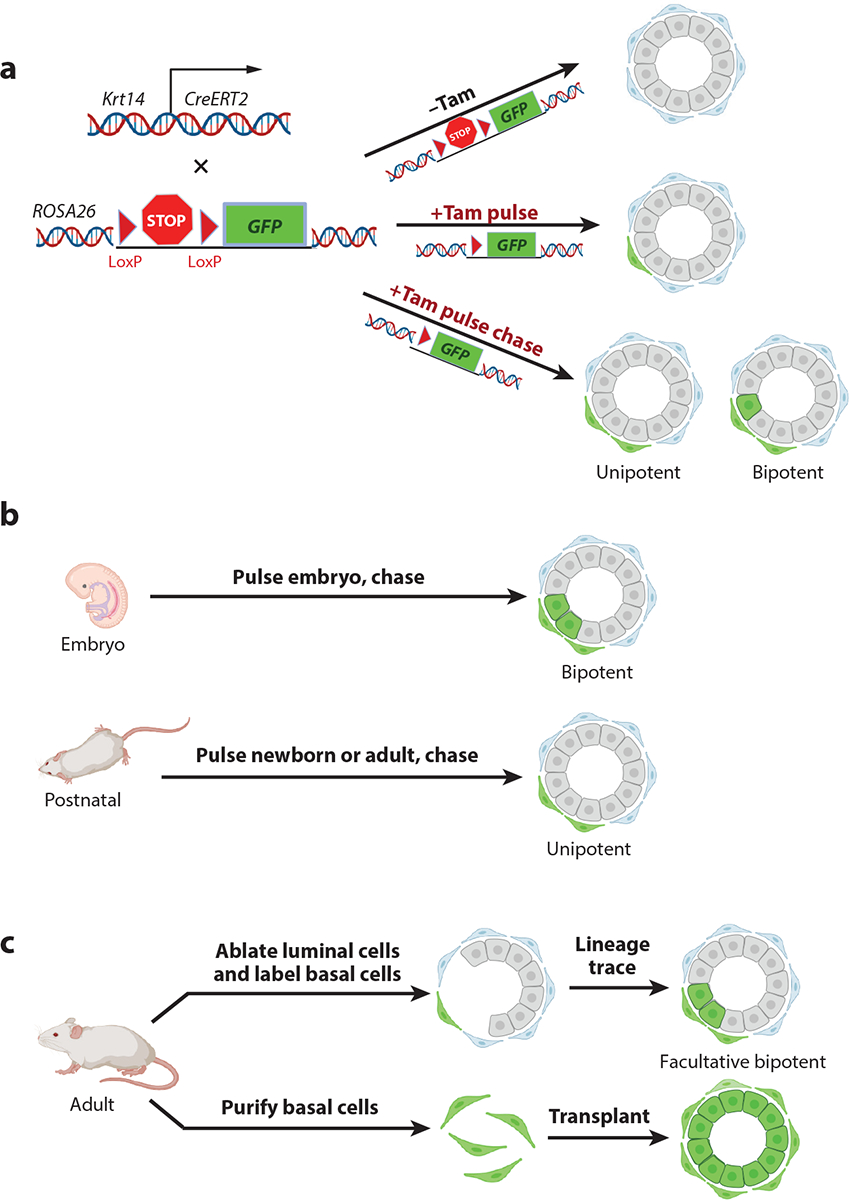
In vivo analyses of cell fate and cell potential. (*a*) Using lineage tracing to determine cell fate in mice. Irreversible cell marking is achieved by expressing a form of Cre recombinase (*CreERT2*) that is inactive in the absence of tamoxifen (Tam) but becomes active upon Tam addition. When combined with a reporter allele embedded in the genome, Cre excises transcriptional stop sequences contained between two Cre recognition sites (loxP sites, represented as *triangles*), resulting in the expression of an easily scored marker such as green fluorescent protein (*GFP*). In this example, mice expressing *CreERT2* under the regulatory control of the cytokeratin 14 (*Krt14*) gene promoter, which is expressed specifically in the basal cells of the mammary gland, are crossed to *ROSA26*-loxP-STOP-loxP-*GFP* reporter mice. Under control conditions (−Tam), no labeling occurs. Treatment with Tam (+Tam pulse) results in permanent labeling of individual basal cells (*green* cell). Over time, the label will appear in the progeny of the cells that were initially labeled (+Tam pulse chase), indicating whether they were able to give rise only to basal cells (unipotent) or to both basal cells and adjacent luminal cells (bipotent). (*b*) Dynamic changes in cell potential in the mammary gland. By applying these labeling techniques, researchers have identified a stage-specific shift in the developmental potential of mammary gland basal cells. Specifically, labeling of basal cells in the embryo revealed them to be bipotent, whereas labeling basal cells postnatally revealed them to be unipotent. (*c*) Developmental potential can vary on the basis of experimental conditions. While these lineage tracing approaches suggest that adult mammary basal cells are unipotent, basal cells can also become bipotent under different circumstances. For example, if one selectively ablates luminal cells (by engineering mice that express the diphtheria toxin receptor in luminal cells and then injecting diphtheria toxin into the nipple), basal cells exhibit bipotent behavior. Similarly, transplantation of purified basal cells generates full and functional mammary glands in which all luminal and basal cells are derived from the transplanted basal cells. Hence, basal cells act as facultative stem cells—cells whose lineage potential expands under wound/wound healing conditions. Figure adapted from images created with BioRender.com.

Lineage tracing is an alternative to transplantation. Some methods permit the labeling of specific cell populations (Figure 1a); others, such as cellular barcoding (43), provide a more generalized way of assessing cell potential. When applied to hematopoietic lineages in vivo, in the context of steady state hematopoiesis, these techniques appear to provide a more nuanced picture of stem cell hierarchies. Specifically, such in situ fate mapping studies have indicated that blood cell development is maintained by low-level involvement of numerous long-lived multipotent progenitors (44, 45). Furthermore, these new systems document an underappreciated diversity of long-term repopulating progenitors in the myeloid arm of the hematopoietic system (although these cells seem to be incapable of serial transplantation) (46). These new tracking methods show that HSCs can undergo cell fate transitions distinct from those predicted by a strict hierarchical model (for reviews, see 37, 47). Discordance between cell potential measured by transplantation assays and cell fate mapped by lineage tracing has also been observed in the hair follicle (48) and mammary gland (see 49, 50). Importantly, these observations indicate that cell fates are not as rigidly encoded in stem cells and progenitors as previously envisioned and that interpretations of experiments involving stem cells are profoundly influenced by the assay used for analysis.

### Stem Cells and Cancer

Discordant findings between transplantation-based and lineage-based methods for assessing stem cell activity have important implications for the cancer field, which has relied primarily on the former to test for stem cell activity. The cancer stem cell (CSC) theory posits that tumors establish a hierarchy with stem-like cells at the apex and more differentiated cancer cells at the base (51, 52). Analogous to HSCs, CSCs are predicted to be rare, slowly dividing, and capable of both multilineage differentiation and self-renewal. It is the CSC that is predicted to be uniquely capable of spawning cancerous variants that contribute to intratumoral heterogeneity. This model is thus distinguishable from a stochastic carcinogenesis model in which cancer cells within a tumor have roughly similar growth and differentiation properties.

This distinction has critical implications for cancer therapy. According to the classical model, therapies that eradicate the greatest number of tumor cells should be those with the greatest long-term efficacy. The CSC model, by contrast, predicts that therapies eliminating bulk tumor cells but not CSCs should have poor long-term efficacy due to CSC-initiated tumor regrowth. The idea that CSCs might, like HSCs, enter dormancy also implies that CSCs may be selectively resistant to drugs that target rapidly dividing cells. However, just as normal stem cells enter and exit dormancy periodically, we expect that the same behavior should pertain to CSCs. Moreover, the ability of cells to undergo epigenetic and transcriptional changes that promote cellular plasticity provides a compelling alternative to CSC-based models to explain intratumoral heterogeneity and maintenance.

**Cancer stem cell (CSC):**

a cell capable of highly efficient tumor formation, typically assessed by transplantation into immunodeficient hosts; note that CSCs so defined are distinct from cells that may initiate a tumor in the first place (the so-called cell of origin) or that perpetuate it in vivo

### Leukemia

Early studies of human leukemia revealed parallels to normal hematopoiesis; even before the availability of cytogenetic and molecular data, diseases such as chronic myelogenous leukemia (CML) were proposed to initiate from a cancerous stem cell (53). Nowell and Hungerford’s finding that virtually all CMLs carry a similar chromosome 9;22 translocation, with cells positive for the Philadelphia chromosome (Ph^+^) (54), strongly suggested that CML is monoclonal and that the translocation occurs in a hematopoietic stem cell. This is consistent with the presence of the 9;22 translocation in the myeloid and lymphoid progeny of a Ph^+^-containing stem cell (55–57). Subsequent mouse studies demonstrated that the protein resulting from the 9;22 fusion gene initiates the mouse version of CML only when introduced into HSCs, a finding that provided further evidence that CML originates in this specialized cellular population (58, 59). Taken together, these data indicate that CML arises from a mutation that either occurs in, or generates, a multipotent HSC variant.

Other types of leukemia such as acute myeloid leukemia (AML) and acute lymphoblastic leukemia may also arise from slow cycling cells that are morphologically distinct from most leukemic cells (60, 61) and that may confer resistance to DNA damage-inducing chemotherapeutic agents (62). These data suggested that, like the hematopoietic system, leukemias could be organized hierarchically. Using the same cell surface markers and strategies used to study hematopoiesis, putative leukemia stem cells have been isolated from AML patients. Specifically, leukemic cells expressing HSC markers (e.g., CD34^+^CD38^−^), as opposed to the bulk of leukemia cells, generated blood cancers in mice after transplantation (with self-renewal inferred by the ability to serially transplant the leukemias from mouse to mouse) (63, 64). More recently, naturally occurring passenger mutations have been used as an in vivo lineage tracer to infer cellular ancestries. Such studies indicate that initiation of these diseases may involve HSCs or multipotent progenitor cells (65, 66).

The interpretation that leukemias originate within a stem or progenitor cell is tempered by several considerations. First, transplantation, especially in the irradiated environment necessary to prepare the environment for the transplanted cells, may elicit cell fate relationships that do not occur under normal (nontransplanted) circumstances. Consequently, the properties of multipotency and self-renewal attributed to putative leukemic stem cells following transplantation may reflect their ability to overcome the selective pressures of the experimental system rather than their normal functions in vivo (a caveat that has even greater implications for CSCs in solid tumors, as discussed below). Second, HSCs may comprise a diverse set of multipotent cells and states whose identity and function may depend upon the niches in which they reside. Consequently, conclusions regarding an HSC origin for all leukemias based on studies employing cell surface markers and transplantation remain fraught. Lineage tracing, utilizing barcoding techniques or other approaches, may provide evidence supporting this viewpoint or, as is becoming clearer from studies of normal hematopoiesis, may provide a more complex and nuanced view of leukemic lineages.

### Solid Tumors

For the reasons noted above, it may be more appropriate to describe cells with tumor-initiating properties in mice as xenograft initiating cells (XICs) (67) rather than CSCs. The latter implies that such cells have differentiation and self-renewal properties in patients, whereas the former more precisely states what is being measured: the ability of cells expressing specific surface antigens or genes to initiate tumor formation under specific transplant conditions.

Studies to identify, isolate, and characterize solid tumor CSCs have generally employed strategies analogous to those used for leukemias and tissue-specific stem cells: cell fractionation based on cell surface markers followed by transplantation. Tumor sphere formation, whereby the ability of various tumor cell subsets to expand in vitro (often using ultralow attachment tissue culture wells), provides a surrogate assay for self-renewal that is amenable to molecular analyses and manipulation. Although tumor sphere assays are widely used because of their simplicity, the utility of this approach is limited by absence of an in vivo microenvironment. Lineage tracing has been used in some studies to infer cell fate under conditions that better preserve cell context and microenvironment (e.g., see 68), and this approach has identified presumptive cancer initiating cells in colon (69, 70), breast (71), prostate (72), pancreas (73), and skin (74).

#### Breast cancer.

Breast cancer was the first solid tumor for which XICs were isolated (75). On the basis of the markers evaluated, XICs were identified as expressing high levels of the hyaluronin receptor, CD44, and low or no CD24 (heat stable antigen). While CD44^+^CD24^low/neg^ breast cancer cells were not rare (11–35% of the cell populations) they had >10× higher xenograft initiating capacity than CD44^+^CD24^+^ tumor cells. The CD44^+^CD24^low/neg^ population was heterogeneous, however, since further fractionation using epithelial specific antigen provided ~50-fold XIC activity relative to unfractionated tumor cells. Multipotency of these XICs was inferred from the fact that CD44^+^CD24^low/neg^ cells generated heterogeneous tumors containing CD44^+^CD24^+^ cells, which was presumed to represent a more differentiated cell population.

Another interpretation, derived from transcriptomic analyses (3, 39), is that these subpopulations reflect differences in epithelial-mesenchymal phenotype rather than the progenitor-progeny relationship implied by a CSC model. As described below, cellular plasticity that enables cells to transition between proliferative epithelial states and motile mesenchymal states confers significant fitness advantages. This provides an alternative to the stem cell model for explaining the emergence of heterogeneous cellular phenotypes in tumors. The observation that breast cancer cells exhibiting more mesenchymal phenotypes correlate with increased XIC activity is consistent with this notion (76–78).

Comparisons of XIC transcriptomes with those of normal adult human and mouse mammary cell populations have consistently shown the strongest relatedness to the transcriptomes of cells identified by transplantation studies as mammary stem cells (MaSCs) (3). MaSCs in the adult mammary gland are basal cells, leading to the proposal that a subset of adult basal cells are MaSCs (79, 80). However, as noted above, transplantation reveals the potential of a cell to exhibit properties it may not possess in its native microenvironment. Indeed, lineage tracing by multiple groups (50) strongly supports the conclusion that adult basal cells are unipotent basal progenitors (Figure 1b) (for an exception, see 81). In contrast, transplantation and lineage tracing studies show that bipotential stem cells reside in the fetal mammary gland (Figure 1c) (39, 82) (for reviews, see 49, 50).

Why, then, do XICs exhibit such a strong transcriptional relatedness to mammary basal cells, and why do mammary basal cells show such exceptional transplant efficiency? We propose that the answer relates to three factors. First, the mammary basal cell epigenome is most like cells of the mid-late mammary embryonic cells with the highest transplantation efficiency (83). Second, recent studies show that local tissue disruption caused by selective luminal cell ablation results in mammary cell reprogramming to a developmentally plastic state (84) (Figure 1c); reprogramming is rapid, as it is apparent within days of transplanting pure basal cells (Q. Vallmajo-Martin, Z. Ma, G.M. Wahl & N. Lytle, manuscript in preparation). Reprogramming of the differentiated cell state also occurs in response to chemotherapy-induced damage but not in response to scalpel-induced abrasions to produce physical wounding (85). Cell-intrinsic and non-cell-autonomous mechanisms may contribute to basal cell plasticity (84, 85). Third, basal cells are likely to express the integrins required to interact with the extracellular matrix present in the transplant microenvironment. Together, these observations indicate that adult basal cells should be considered as facultative stem cells that are able to rapidly reprogram their transcriptomes and epigenomes in the wound environment associated with transplantation.

**Facultative stem cells:**

differentiated cells that lack stem cell properties under normal circumstances but can behave like a stem cell under exigent circumstances (such as during tissue repair or transplantation or after therapy-induced tissue damage)

#### Colorectal cancer.

The colon is a dynamic organ in which epithelial cells renew every 3–5 days (86). A common theme regarding homeostasis, injury repair, and carcinogenesis is the role of colon stem cells and the ability (through plasticity) of differentiated cells to reprogram to a stem-like state. Unlike the mammary gland, the intestine contains at least two types of adult tissue-resident stem cells that are distinguishable from each other positionally, molecularly, and functionally (87, 88). The most well-characterized intestinal stem cells are mitotically active pyramidal-shaped cells located in the base of each crypt that express Wnt-driven genes such as the R-spondin receptor *LGR5* and *EPHB2* (86, 89). These crypt base columnar (CBC) cells were shown by lineage tracing to generate all differentiated colon cell types (absorptive enterocytes and secretory endocrine cells, goblet cells, and Paneth cells) and to self-renew. However, the intestine maintains a reserve stem cell system, as selective CBC ablation enables recruitment of other cells, including those with a more differentiated phenotype, to perform stem cell functions (90, 91).

Colon regeneration after CBC ablation revealed that differentiated cells can undergo adaptive reprogramming to enable damage repair. For example, helminth infection and consequent intestinal wall disruption generate granulomas at the crypt base and loss of Lgr5^+^ CBC stem cells. In that setting, Lgr5-negative differentiated cells reprogrammed into a fetal-like state via T cell–mediated secretion of IFN-γ (92). Likewise, disruptions of tissue integrity result in YAP pathway activation and reacquisition of a fetal-like gene signature, suggesting that alterations in mechanical signaling trigger cell state reprogramming to reestablish tissue integrity (93). These studies indicate that there are two types of intestinal stem cells: (*a*) CBC stem cells, which are Lgr5-positive and dominate under conditions of normal homeostasis, and (*b*) facultative or regenerative stem cells, which are Lgr5-negative but capable of reprogramming to an Lgr5^+^ stem-like state following injury.

The studies summarized above have indicated how CSCs may contribute to colorectal cancer. Using the now-standard transplantation model, the cell surface markers CD133 and CD44 have been used to fractionate human colorectal cancers into subpopulations to identify subpopulations with high XIC efficiency (94–96). These studies showed that cells expressing high levels of CD133 and/or CD44 exhibited significantly higher XIC efficiency than did cells expressing low levels of these markers, with tumors exhibiting marker heterogeneity similar to that present in the original tumors (94). Significantly, just a single CD44^+^ cell from a human tumor could be xenografted subcutaneously to produce a heterogeneous tumor containing the same cell types as found in the normal colon (96).

As discussed above, transplantation approaches may elicit cellular potential not present in vivo. Hence, it is not clear whether the XICs identified in the above studies reflect stem cell activities present inside actual human tumors. Moreover, the extraordinary plasticity present in the normal intestine creates complications for the CSC model, especially if differentiation states in colorectal cancer are as plastic as those that present normally. If differentiated progeny can assume stem cell activity under benign conditions, what would prevent a similar change in cell state from happening under malignant conditions? Stated otherwise, the extensive cellular plasticity characteristic of tumors (detailed further below) confounds our understanding of the role and even the existence of CSCs.

### Other Thoughts Regarding Cancer Stem Cells

It is worth making two final points regarding the assays used to study putative CSCs. First, it is important to emphasize that most studies that have identified XICs have not used transplantation with cancer-associated fibroblasts or other stromal components that coevolve during tumor development and have been shown to contribute significantly to tumor-initiating ability (97, 98). It is possible that inclusion of such components would yield XICs with different cell surface and molecular characteristics. Second, there is experimental evidence that XIC frequency is far greater than that suggested by standard transplantation assays. In melanoma, for example, altering the host or the transplant method to create conditions more conducive for tumor growth—reducing immune surveillance or providing a tissue scaffold—caused the fraction of tumor-initiating cells to increase from ~0.0001% of tumor cells to >10% of tumor cells (99). Similarly, changing the conditions under which assays used to detect leukemic stem cells were performed led to the conclusion that 10% or more of the cells in a murine leukemia model exhibited tumor-initiating properties (100). These properties—that the efficiency of tumor initiation following transplantation depends heavily on host factors—may or may not extend to other tumor types. Nevertheless, these findings suggest that the ability to propagate a tumor may not be limited to a very rare population of cells but is instead a broadly shared property of all cancer cells.

## DIFFERENTIATION, PLASTICITY, AND CANCER

### Cancer Is a Disease of Altered Cellular Differentiation

**Figure 2:**
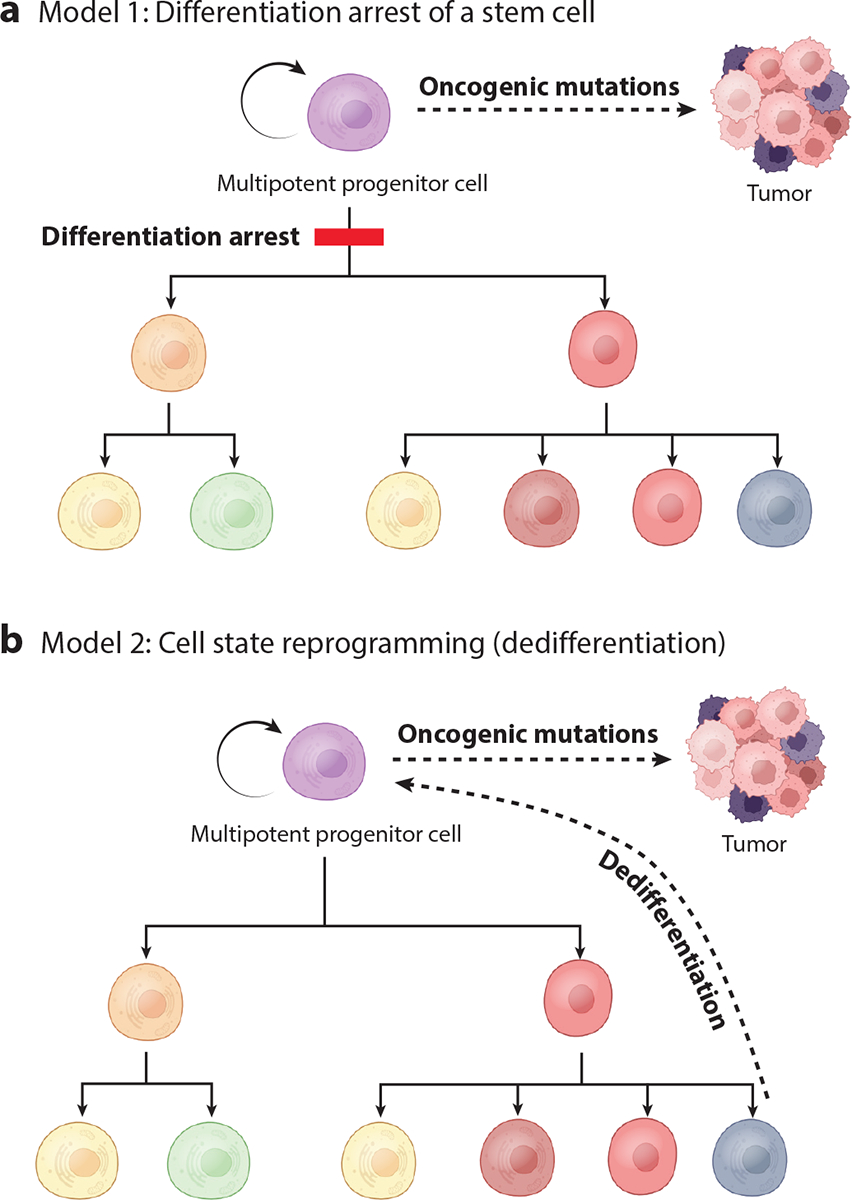
Models for tumor progression via differentiation arrest versus cell state reprogramming. (*a*) Model 1 indicates that mutations and/or epigenetic events serve to arrest cells at an early intermediate state of differentiation, resulting in accumulation of proliferative multipotential progenitor cells or stem cells. Subsequent transforming events stimulate proliferation and evolution to a tumor with cells exhibiting multiple degrees of cellular differentiation. According to this model, the dedifferentiated components of a tumor derive from its less-differentiated origins. (*b*) Model 2 indicates that mutations and/or epigenetic events serve to drive cells to regain a proliferative progenitor cell phenotype. Again, subsequent transforming events promote evolution to a tumor with multiple degrees of cellular differentiation. In this case, the dedifferentiated components are the result of a dedifferentiation event. Figure adapted from images created with BioRender.com.

Given that the many tissues of the body are all derived from a single cell, normal embryonic development necessitates both a quantitative increase in the number of cells (proliferation) and a qualitative diversification of cellular phenotypes (differentiation). Differentiation manifests itself in the form of histologically distinguishable cell types bearing morphological features and gene and protein expression patterns that subserve function. Cancers typically have a histologically dedifferentiated appearance, a term that implies that tumor cells lose their differentiated features. While the absence of such features may reflect a true loss of the differentiated state (with examples from breast cancer provided below), the absence of differentiated features could also reflect a failure to acquire such features in the first place. This latter possibility could hold in cases where tumors arise from tissue-resident stem cells that lack specialized characteristics (Figure 2). In either case, the degree of differentiation in a tumor carries important prognostic information. A tumor’s differentiation status is known as tumor grade, and poorly differentiated tumors (i.e., tumors that are highly disorganized, lack normal structures, and have highly atypical nuclei) carry a poor prognosis regardless of tumor type or stage.

It is unknown whether this relationship between tissue level changes in differentiation and cancer is merely correlative. However, several lines of evidence suggest that dedifferentiation may functionally contribute to tumor progression. A classic example is acute promyelocytic leukemia (APL), the result of a translocation that creates a retinoic acid receptor alpha (*RAR*α) fusion gene. APL cells resemble myeloid progenitor cells, the less-differentiated cell population that normally resides in the bone marrow. Remarkably, treatment regimens that include all-*trans* retinoic acid (ATRA) (101)—the ligand for the RARα receptor—cause leukemic cells to differentiate and are associated with excellent clinical responses (102). Dedifferentiation seems to be a requisite feature of tumor progression in other tumor types as well. For example, loss of *PTF1A*, a master regulator of pancreatic acinar differentiation, is a rate-limiting step for pancreatic tumor development (103), while mutations in *IDH* promote cholangiocarcinoma by interfering with the differentiated state of hepatocytes (104).

If a terminally differentiated state is incompatible with malignant growth, then why hasn’t this paradigm yet translated from APL to other leukemias and solid tumors? One possibility is that cellular differentiation and clinical response in APL, while correlated, may not be causally related. For example, therapies other than ATRA are capable of inducing the differentiation of leukemic cells without prompting a clinical remission (105), a result that may suggest that the *RARα* fusion gene regulates genetic programs that independently govern cell growth and cell differentiation programs. If this is true, then any therapy that targets a driver oncogene may result in apparent tumor differentiation, as has been observed in osteogenic sarcomas following the silencing of the *MYC* oncogene (106) and in glioblastomas following treatment with inhibitors of mutant *IDH* (107). Alternatively, APL may be an outlier on the basis of its unique genomic features. As the arsenal of drugs with anticancer activity continues to grow, we will learn whether prodifferentiation agents can act independently of their effects on tumor growth programs or whether differentiation is merely a biomarker of an effectively targeted genetic driver.

### Epithelial Plasticity and Epithelial-to-Mesenchymal Transition

Cell state plasticity, or cellular reprogramming, describes the processes by which cells undergo dramatic changes in identity or phenotype with associated genetic, epigenetic, and/or transcriptional alterations (dedifferentiation is one example). Plasticity is a hallmark of embryonic development, as cells require great flexibility to properly assemble into tissues, and it is now being recognized as a hallmark of cancer (9). One of the best studied examples of cellular plasticity concerns the interconversion of cells with an epithelial versus a mesenchymal phenotype. First described by Elizabeth Hay in the context of normal development as an epithelio-mesenchymal transformation (108), the processes we now refer to as epithelial-to-mesenchymal transition (EMT) and mesenchymal-to-epithelial transition (MET) are common in malignancy (109).

In carcinomas, which arise within the epithelial compartment of endoderm- and ectoderm-derived tissues, EMT is closely associated with tumor grade. In well-differentiated or low-grade tumors, most cancer cells retain an epithelial phenotype. In poorly differentiated or high-grade tumors, by contrast, most cancer cells have lost their epithelial characteristics—often resembling cells of a mesenchymal lineage such as fibroblasts. These phenotypic changes come about through a diverse set of EMT/MET programs and represent an important prognostic indicator across tumor types.

An important function of EMT in development is to equip cells with the capacity to move. Cells residing within an epithelial layer are relatively immobile, a consequence of the adherens junctions and tight junctions that bond them to their neighbors. But during organ formation, cells must undergo dramatic movements, and epithelial plasticity allows them to do so. In carcinomas, this property facilitates the ability of cancer cells to invade, enter the bloodstream, and give rise to metastases (110). In addition to the wealth of evidence suggesting that EMT promotes the early steps in metastatic spread, there is also evidence that the reverse process promotes outgrowth (colonization) at distant sites (111, 112). EMT and MET (or, more generally, epithelial plasticity) result in a broad rewiring of cellular mRNA and protein composition. Consequently, EMT has been associated with resistance to chemotherapy in both preclinical and clinical settings (113).

Cancer biologists have yet to fully exploit the therapeutic opportunities associated with EMT. The molecular rewiring that affords cells with resistance to certain drugs likely confers new vulnerabilities that can be exploited with other agents. In pancreas cancer, for example, cell lines that have mesenchymal features tend to be resistant to inhibition of the epidermal growth factor receptor (EGFR) but exhibit sensitivity to gemcitabine chemotherapy (114). Likewise, EMT appears to result in metabolic shifts that can create new therapeutic vulnerabilities (115). A further understanding of the vulnerabilities that are newly revealed by EMT may enable combination strategies that target both the epithelial and mesenchymal subpopulations within a tumor.

The molecular mechanisms underlying cellular plasticity are still being worked out. Nevertheless, it is likely that overlapping molecular mechanisms drive EMT and MET in embryonic development and cancer. The most well-characterized plasticity programs are those that operate at the level of gene transcription, where so-called EMT transcription factors (EMT-TFs) are responsible for repressing genes associated with the epithelial state and activating genes associated with the mesenchymal state. Genes such as *SNAIL* and *TWIST*—which are necessary for certain EMT-associated events in vertebrate embryos (e.g., gastrulation)—are archetypes of this class of EMT-TFs; these and other transcription factors also play a role in cancer-associated EMT (116). However, cells may also shed their epithelial phenotype by posttranscriptional means, which may alter their invasive behaviors from single-cell to collective migration (117).

These distinct and overlapping EMT programs generate cells possessing a wide range of phenotypes, resulting in a spectrum of partial-EMT states whose behaviors and molecular features are absent from cells residing at either extreme of the continuum (fully epithelial or fully mesenchymal) (118). Barcoding studies in pancreas cancer suggest that this variation in epithelial-mesenchymal states arises stochastically (119). Thus, a cell’s position along the epithelial-to-mesenchymal continuum may provide an advantage when certain selective conditions are applied, such as the ability to enter the bloodstream or survive chemotherapy.

**Epithelial-to-mesenchymal transition (EMT):**

a form of plasticity by which epithelial cells acquire features of mesenchymal cells such as fibroblasts or leukocytes; related to the reverse process by which mesenchymal cells acquire epithelial features (MET)

### Cell State Plasticity and Cancer Origins

Because cancer is a result of both genetic and epigenetic alterations, the epigenetic makeup of the cell(s) from which a tumor derives has important implications for its biology. Indeed, a comparison of different schemes for classifying tumors found that a tumor’s tissue of origin dominates (26), in line with the anatomical taxonomies that oncologists and pathologists have used for more than a century. Within a given tissue, however, the ability of cell types to reprogram and interconvert makes it difficult to ascertain the cell of origin for many cancers. For example, breast cancers in *BRCA1* mutation carriers are typically high-grade, invasive ductal adenocarcinomas that fall into the triple-negative group (i.e., they lack estrogen and progesterone receptor expression and do not exhibit Her2 amplification). Although the histological appearance of such tumors would suggest that they originate from mammary basal cells, experimental evidence in mouse models suggests otherwise. Specifically, deleting *BRCA1* in murine luminal cells, but not basal cells, generates tumors resembling human basal-like breast cancers (101). Importantly, such a discordance between histological appearance and experimentally determined cell of origin is not limited to breast cancer (120).

**Metaplasia:**

the replacement of one histological cell type with another, often occurring in association with injury or inflammation and representing a harbinger of cancer development

**Figure 3:**
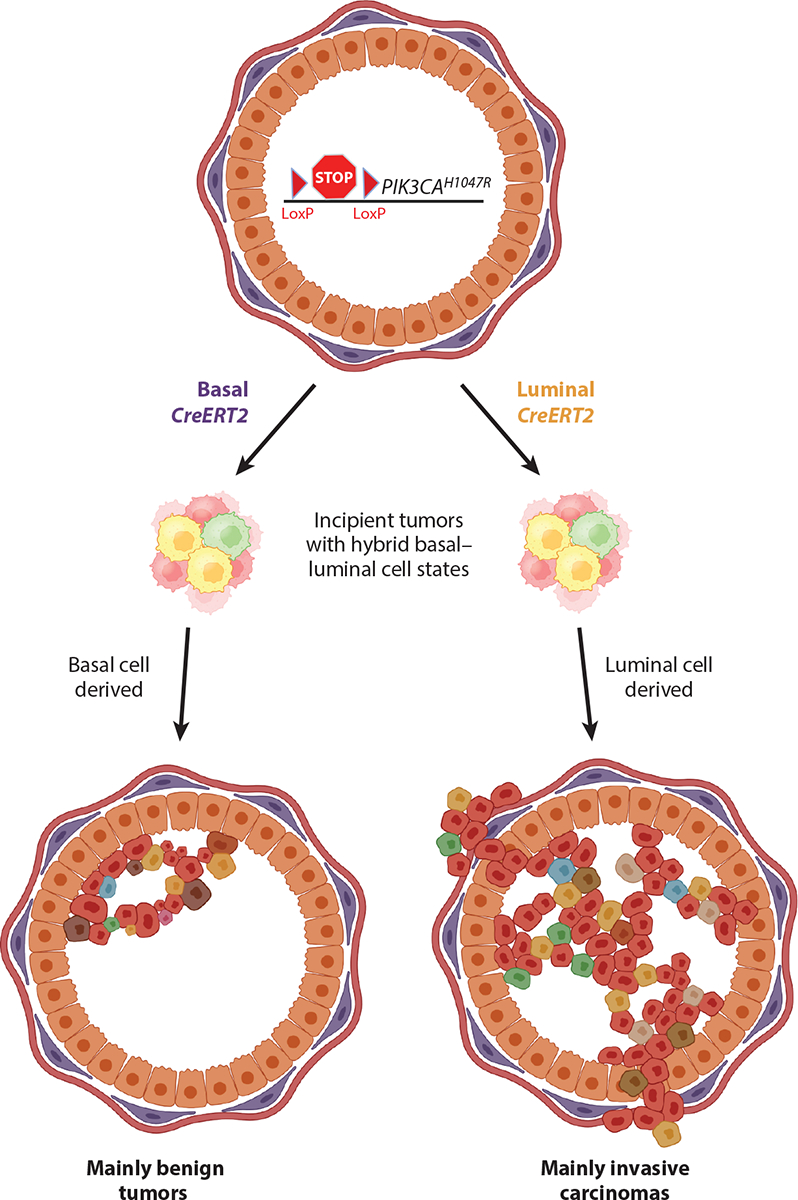
Oncogene activation in different types of mammary cells results in tumors with differing aggressiveness. Mice engineered to express a conditionally activatable form of oncogenic phosphoinositide 3-kinase (*PIK3CA*^*H1047R*^) can be crossed with mice expressing a Tam-inducible Cre (*CreERT2*) under the control of a (*left*) basal or (*right*) luminal promoter. In both cases, early steps in tumor formation involved reprogramming of basal and luminal cells to a mixed/hybrid cell state. Subsequently, tumors arising from this hybrid cell state behaved differently depending on whether the oncogene was initiated in basal or luminal cells. Whereas tumors arising from basal cells were largely benign, tumors arising from luminal cells were highly aggressive and had transcriptomes corresponding to a variety of human breast cancer subtypes. Such findings underscore the importance of the cellular origins of a tumor in its late-stage biology (and highlight the difficulty of inferring the cell of origin on the basis of the histology or transcriptome of the resulting tumor). Figure adapted from images created with BioRender.com.

How does such a histological transformation take place? A time-resolved analysis of luminal cells engineered to simultaneously lose *BRCA1* and *TP53*, as commonly observed in human basal-like tumors, revealed features of aberrant differentiation (i.e., dedifferentiation) (121). Other studies showed that expressing a constitutively active *PI3K* allele (*PI3K*^*H1047R*^) frequently found in human breast cancers in either mouse basal or luminal cells induced reprogramming to a hybrid cell state resembling basal-like human breast tumors. In this case, the cell of origin impacted the final molecular subtypes and aggressiveness of the resulting tumors (122, 123) (Figure 3). Inactivating mutations in the COMPASS histone methyltransferase complex, which occur in 40% of human breast cancers, can cooperate with activating PI3K mutations to also elicit aberrant differentiation programs; in this case, however, these mutations promote the genesis of luminal-like tumors from basal cells (124). Together, these studies emphasize the importance of cell state reprogramming in adult, differentiated cells, not stem cells, in the genesis of heterogeneous tumors comprising all breast cancer subtypes.

A similarly counterintuitive relationship between cell of origin and tumor histology exists in the pancreas, where pancreatic ductal adenocarcinoma (PDAC) would logically be assumed to arise from the ductal compartment of the pancreas. Contrary to this expectation, several studies in mice and humans indicate that the acinar cells of the pancreas—the much larger cellular compartment responsible for making the organ’s digestive enzymes—serves as a major source of most pancreatic tumors. Here, a different form of plasticity known as metaplasia is responsible for the histological makeover. Metaplasia describes the replacement of cells of one type with cells of another type and commonly precedes the development of carcinomas of the esophagus, stomach, and cervix (125). In the pancreas, a so-called acinar-to-ductal metaplasia is believed to facilitate malignant transformation by creating a unique epigenetic state (126, 127). Thus, it is likely that multiple cellular compartments can give rise to PDAC but take different molecular paths to reach similar end points.

A similar ambiguity over cancer origins has played out in the colon, where it appears that colon cancers can initiate from both stem and differentiated cells. Importantly, cell type profoundly influences the types of mutations and conditions required for cancer initiation and progression. For example, precancerous tumors (adenomas) can arise either in the crypt base where the Lgr5^+^ CBC stem cells reside or in differentiated villus cells. A significant fraction of microadenomas in humans frequently arise at the top of colonic glands with no clear connection to the stem cell crypt (128). This suggests at least two mechanisms for colon carcinogenesis, one involving stem cells in the crypt base, referred to as bottom up, and the second involving initiation in transit-amplifying or differentiated cells at higher levels of each villus (top down). Each example involves initiation by different mechanisms, with distinct initiating oncogenes and inflammation playing an important role in cellular reprogramming of differentiated villus cells to a stem-like state (129). We speculate that, as in normal tissue repair, conditions that perturb morphogen gradients (130) or result in mechanical stresses caused by disruption of tissue integrity (93) may trigger cell state reprogramming during cancer progression.

The ability to track tumor evolution from a single normal cell to metastatic variants is now possible due to advances in CRISPR-based barcoding techniques. In lung cancer, for example, computational analysis of the fluorescently labeled, barcoded tumor cells enabled phylogenies to be deduced and estimates of clonal fitness to be generated. The data revealed that activation of *KRAS* and loss of *TP53* in an alveolar type 2 (AT2) cell initiate tumor formation through a transient increase in cellular plasticity, involving gastric-like or lung-mixed states followed by EMT-associated intermediates, before subclones capable of metastasizing arise (131). Loss of additional tumor suppressors including *LKB1* or *APC* increased the rate of tumor progression, altered the evolutionary trajectories, and increased fitness of some subclones. (Here, fitness means the ability of individual cells to drive tumor expansion through acquisition of specific fitness signatures that correlate with the probability of clonal expansion.) It is noteworthy that the cells with the lowest fitness-signature score were the most AT2-like (most differentiated), while those with the highest fitness score correlated with a mesenchymal state derived from EMT-like cells. Importantly, the fitness-signature scores correlated significantly with prognosis for patients with lung adenocarcinoma. Thus, the acquisition of developmentally plastic intermediate states appears to be an important step in the development of greater fitness states that enable cancer cells to grow and spread.

### Other Forms of Plasticity

Changes in cell state other than shifts between epithelial and mesenchymal phenotypes contribute further to tumor aggressiveness or therapy resistance. The two most prominent examples are the neuroendocrine-like states adopted by lung and prostate tumors that have overcome therapies that target EGFR or the androgen receptor (AR), respectively. In the case of the lung, as many as 14% of non-small cell lung cancers acquire resistance to EGFR inhibitors by adopting a small cell lung cancer (SCLC) neuroendocrine-like phenotype (132). Molecular analysis of this phenomenon suggests that the transformation is almost always associated with de novo mutations in the retinoblastoma (*RB*) tumor suppressor gene and frequently associated with mutations in the p53 (*TP53*) tumor suppressor. Importantly, these mutations are likely necessary but not sufficient for the transition to SCLC (133). A similar story has played out in prostate cancer, where as many as 20% of patients with resistance to AR blockade exhibit a neuroendocrine-like histology and associated molecular features; again, these tumors are associated with acquired mutations in *RB* and *TP53* (134). At present, it is unknown how these mutations contribute to histological transformation or how such alterations in cell state lead to resistant phenotypes.

**Embryonic induction:**

the developmental process by which one group of cells directs the development of another group of cells

## CELLULAR CROSS TALK

Embryonic induction describes the process whereby groups of cells in the developing embryo become signaling hubs to alter the fate, position, or behavior of neighboring cells. Induction, and the resulting cascade of reciprocal signaling, ensures that tissues achieve their appropriate cellular makeups and three-dimensional configurations. Thus, the distinctive architecture of every tissue—from the villi of the small intestine to the branched ducts of the mammary gland—can trace its origins back to these initial intercellular conversations. Tumor cells, along with the diverse cells and matrix elements that make up the tumor microenvironment (TME), engage in similar intra-cellular cross talk during tumor progression, often using the same signaling molecules employed by the embryo. However, whereas the histological outcomes of embryonic signaling events are easily recognized in normal tissues, with their stereotypic architecture, the signaling events guiding the spatial organization of a tumor are harder to deconvolute. Given the growing interest in targeting the tumor stroma in addition to tumor cells (135), it will be critical to understand the molecular logic underlying TME organization.

### Tissue Organizers

The most famous example of tissue induction is the Spemann–Mangold organizer, a collection of embryonic cells responsible for inducing the nervous system. As such, the cells making up the Spemann–Mangold organizer hold a specialized place at the apex of a hierarchy of embryonic signaling. Similarly, in tumors, cancer cells sit at the top of an organizational hierarchy, dictating the makeup of their surrounding microenvironment. Such tumor organizers may shape the makeup of the tumor during its initial growth stages or during metastasis, when disseminated cancer cells must establish a new microenvironment (136).

Several studies of the TME—particularly its immune composition—have been useful for delineating the actions of tumor organizers. By recognizing unfamiliar antigens that invariably emerge during tumor progression, the immune system exerts a strong selective pressure that cancer cells must circumvent. Some cancer cells achieve this through immunoediting, a sculpting process whereby cancer cells manage to evade immune cell recognition and/or killing (137). A classical example of this comes from melanoma patients, where the MART-1 antigen is lost from cancer cells following the infusion of MART-1-specific T cells (138). But cancer cells can also avoid immune destruction by creating microenvironments that prevent the entry or activity of cytolytic T cells, a feat they accomplish by secreting chemokines that recruit immunosuppressive myeloid cells and macrophages (139). Other features of the TME such as cancer-associated fibroblasts, tumor vasculature, and matrix are likely to be under the influence of similar organizer-like activities. In tumors, therefore, cancer cells are the architects of their microenvironments, just as their embryonic counterparts are responsible for shaping tissues and organs.

The organizer concept helps explain how TMEs are established in the first place, at early stages of tumor development (or metastasis). But as tumors grow, two things happen: (*a*) tumors evolve, causing cancer cells in different parts of a tumor to have distinct genetic and epigenetic makeups, and (*b*) cells in the microenvironment begin to have their own conversations without the input of tumor cells. At these later stages, cellular cross talk becomes much harder to deconvolute, as multiple signals from multiple sources can be sensed and acted upon by multiple recipients. Again, the signals and the molecular logic that guides the morphogenetic movements of these divergent cell types during normal development are likely reused by both tumor cells and stromal cells during the evolution of the TME, with its many neighborhoods.

We have much to learn about these complex cellular conversations, but a few examples stand out. For instance, sonic hedgehog (SHH) produced by embryonic epithelial cells is known to act on adjacent mesenchymal cells, leading to the formation of subepithelial tissue layers such as the submucosa of the gut. In cancer, such paracrine SHH signaling promotes the outgrowth of the surrounding stroma (140). Accordingly, ablation of SHH in pancreatic tumor cells results in a depletion of cancer-associated fibroblasts, with secondary effects on other stromal cell types (141). Similarly, GM-CSF (granulocyte-macrophage colony-stimulating factor) produced by pancreatic cancer cells stimulates the recruitment of myeloid cells, contributing to the immunosuppressed TME of this cancer type (142, 143). Because tumors lack the stereotyped cell–cell interactions imposed by embryogenesis, the complexity of signaling in a tumor likely far exceeds that present in normal developing tissues. Nevertheless, recent advances in computational biology and single-cell sequencing may help cancer biologists infer the molecular mediators of intratumoral cross talk in the future (126).

## CONCLUSIONS AND FUTURE DIRECTIONS

There are numerous similarities between tumors and embryos. Both rely on the cell cycle and stem cell programs to fuel massive cell proliferation, cellular plasticity to achieve a diversity of cellular states, and signaling cross talk to build functional microenvironments. Evolution defines both processes. In the context of development, millennia of evolutionary pressure shaped the consistent patterns defining the growth and form of normal tissues. Cancers, by contrast, co-opt those same evolutionary forces to obtain a selective advantage. This does not mean that cancer cells invent new biology, creating molecular pathways that are unknown to the organism. Rather, it is our view that cancer cells use existing biology in creative ways, with much of that biology derived from normal embryological processes.

There are several ways in which understanding the developmental roots of tumors could help cancer biologists in the future. The first is a better understanding of cellular plasticity. Learning the path(s) that various cell types follow en route to malignant transformation—whether stem cells or differentiated cells—may help us understand the relationship between baseline epigenetic landscapes and subsequent oncogenic events, paving the way for new differentiation therapy strategies. Likewise, epithelial plasticity (EMT and MET) plays a role in both metastasis and chemoresistance, yet most of our understanding of how these programs operate in vivo derives from studies of embryonic plasticity programs (e.g., gastrulation and neural crest migration). Given the extraordinary complexity of cell states in cancer, much can still be learned by studying comparatively simpler examples of cellular plasticity in the embryo. Finally, there is an increasing emphasis on developing strategies that disrupt the tumor stroma—fibroblasts, endothelial cells, immune cells, and matrix—in combination with other anticancer therapies, a goal that will require a detailed understanding of the signals shaping the TME. Given that stromal cells are nontransformed, their behavior within the TME is almost certainly guided by the same logic that guided their activity in the embryo.
